# *In vitro* anti-*Helicobacter pylori* activity and the underlining mechanism of an empirical herbal formula – Hezi Qingyou

**DOI:** 10.3389/fmicb.2024.1355460

**Published:** 2024-02-19

**Authors:** Zhong Feng, Hui Li, Yajie Hao, Chang Peng, Ling Ou, Junwei Jia, Mingjin Xun, Yuanjing Zou, Meiyun Chen, Guimin Zhang, Meicun Yao

**Affiliations:** ^1^School of Pharmaceutical Sciences, Sun Yat-sen University, Shenzhen, China; ^2^State Key Laboratory of Integration and Innovation of Classic Formula and Modern Chinese Medicine, Lunan Pharmaceutical Group Co., Ltd., Linyi, China; ^3^International Pharmaceutical Engineering Laboratory in Shandong Province, Shandong New Time Pharmaceutical Co., Ltd., Linyi, China; ^4^Nanchang Research Institute, Sun Yat-sen University, Jiangxi, China

**Keywords:** *Helicobacter pylori*, Hezi Qingyou Formula, antibacterial activity, mechanism of action, *in vitro*

## Abstract

**Background:**

*Helicobacter pylori* (*H. pylori*) is thought to primarily colonize the human stomach and lead to various gastrointestinal disorders, such as gastritis and gastric cancer. Currently, main eradication treatment is triple or quadruple therapy centered on antibiotics. Due to antibiotic resistance, the eradication rate of *H. pylori* is decreasing gradually. Therefore, searching for anti-*H. pylori* drugs from herbal sources has become a strategy for the treatment. Our team proposed a Hezi Qingyou Formula (HZQYF), composed of Chebulae Fructus, Ficus hirta Vahl and Cloves, and studied its anti-*H. pylori* activity and mechanism.

**Methods:**

Chemical components of HZQYF were studied using UHPLC–MS/MS and HPLC. Broth microdilution method and agar dilution method were used to evaluate HZQYF’s antibacterial activity. The effects of HZQYF on expression of adhesion genes (*alpA*, *alpB*, *babA*), urease genes (*ureE*, *ureF*), and flagellar genes (*flaA, flaB*) were explored using Reverse Transcription-quantitative Polymerase Chain Reaction (RT-qPCR) technology. Effects on morphology and permeability of the extracellular membrane were studied using scanning electron microscopy (SEM) and N-phenylnaphthalen-1-amine (NPN) uptake. Effect on urease activity was studied using a urease kinetics analysis *in vitro*. Immunofluorescence staining method was used to examine the effect on adhesion. Western blot was used to examine the effect on cagA protein.

**Results:**

Minimum inhibitory concentration (MIC) values of the formula against *H. pylori* clinical strains and standard strains were 80–160 μg/mL, and minimum bactericidal concentration (MBC) values were 160–320 μg/mL. The formula could down-regulate the expression of adhesion genes (*alp*A, *alpB*, *babA*), urease genes (*ureE*, *ureF*) and flagellar genes (*flaA*, *flaB*), change the morphology of *H. pylori*, increase its extracellular membrane permeability, and decrease its urease activity.

**Conclusion:**

Present studies confirmed that HZQYF had promising *in vitro* anti-*H. pylori* activities and demonstrated its possible mechanism of action by down-regulating the bacterial adhesion, urease, and flagellar gene expression, which provided scientific bases for further clinical investigations.

## 1 Introduction

*Helicobacter pylori* (*H. pylori*) is a Gram-negative microaerobic bacterium with a flagellum and multiple virulence factors. Currently, *H. pylori* infects more than half of the world’s population, with an infection rate of 56.22% in China (Nagy et al., 2016). *H. pylori* infection has been linked to a variety of gastrointestinal and extra-gastrointestinal diseases, including adverse metabolic characteristics in obese people, type II diabetes, ischemic heart disease, anemia, gastric ulcers, etc. (Bravo et al., 2018). Research indicated that eradicating *H. pylori* infection can prevent the development of various gastric diseases (Hu et al., 2017), such as peptic ulcers (Liu et al., 2017) and gastric cancer (Li et al., 2021). Currently, antibiotics are used in the majority of *H. pylori* infection treatment regimens, including standard triple and bismuth quadruple therapy (Malfertheiner et al., 2016). However, the worldwide abuse of antibiotics has led to the growth of *H. pylori*’s resistance toward commonly used antibiotics, resulting in a significant obstacle in the successful treatment of *H. pylori* infection (Thung et al., 2016). Previous studies indicated that a significant proportion of treatments, ranging from 10 to 30 percent of the initial and subsequent treatment plans proposed in global treatment guidelines have been proven ineffective over the past decade (Li et al., 2015; Malfertheiner et al., 2016). In instances where the antibiotics-centered treatment fails to achieve a cure of *H. pylori* infection, the absence of any reliable therapeutic alternatives poses considerable challenges for patients in terms of their treatment options (Shiotani et al., 2017; Fallone et al., 2019). Searching for anti-*H. pylori* drugs from herbal medicines has been recommended as an effective strategy (Ghasemian et al., 2019). There are various herbal medicines, such as *Coptis chinensis* Franch (Huanglian) and *Scutellaria baicalensis* Georgi (Huangqin), showing potent anti-*H. pylori* activity (Ma, 2010). However, their primary antimicrobial ability lies in direct bactericidal action, which is similar to the action of antibiotics. Therefore, despite their strong bactericidal capabilities, prolonged use of these medicines can also lead to toxic side effects such as diarrhea. Consequently, it is more beneficial to look for Chinese herbal medications with improved anti-*H. pylori* activities and decreased side effects.

In addition, the integration of traditional Chinese medicine with antibiotics has emerged as a novel approach in antimicrobial therapy. Extensive research has demonstrated that the concurrent administration of traditional Chinese medicine and antibiotics can significantly enhance the eradication efficacy of *H. pylori* when compared to the conventional clinical antibiotic triple or quadruple therapy (Xue et al., 2023).

We have discovered a Chinese medicine formula from clinical practice, which had effectively reduced the load of *H. pylori* in the stomach of infected individuals. Furthermore, it was found in long-term clinical observations that patients using this formula indeed experienced very few side effects such as diarrhea. The formula is composed of Chebulae Fructus, Ficus hirta Vahl, and Cloves, which is called “Hezi Qingyou Formula (HZQYF).”

Chebulae Fructus, also known as Shijunzi or Rongmao Chebulae Fructus, refers to the ripe dried fruit of the plant belonging to the Rutaceae family. Chebulae Fructus is listed in various editions of the “Pharmacopoeia of the People’s Republic of China.” Our team has discovered that Chebulae Fructus exhibits a good anti-*H. pylori* effect *in vitro* (Ou et al., 2023), with an MIC for antibacterial activity at approximately 20–160 μg/mL.

Ficus hirta Vahl, also known as Five Fingered Gourd, is the root of *Ficus simplicissima* Lour., a plant of the mulberry family (*Moraceae*). Research indicates that the water extract of Ficus hirta Vahl inhibits the growth of some common bacteria, such as *Escherichia coli*, *Bacillus subtilis*, and *Staphylococcus aureus*. Modern pharmacology indicates that Ficus hirta Vahl has several beneficial effects, including enhancing the immune system, antibacterial properties, protecting the gastric mucosa, improving microcirculation, and acting as an antioxidant (Au, 2009; Zeng et al., 2012; Ma et al., 2017). Therefore, in the formulation of HZQYF, the role of Ficus hirta Vahl in terms of antibacterial action is not the primary focus, and enhancing immune function should be the main consideration.

Cloves refer to the dried flower buds of the plant *Eugenia caryophyllata Thunb.* from the *Myrtaceae* family. As a traditional Chinese medicine, cloves are used to alleviate symptoms such as spleen and stomach ailments, belching, and vomiting. Recent research has shown that cloves have various potential therapeutic properties, including antiviral, antifungal, anticancer, antioxidant, and anti-inflammatory effects (Zaidi et al., 2012). Our previous research indicated that cloves exhibit promising anti-*H. pylori* activity (Peng et al., 2022). They also show potential for alleviating inflammation caused by *H. pylori* infection and enhancing the innate immune system’s function (Peng et al., 2023). Cloves in the HZQYF may possess both antibacterial effects and the ability to ease gastrointestinal symptoms, as well as enhance host innate immunity.

Among the three herbs in the HZQYF, Chebulae Fructus not only has a strong bactericidal effect but also exhibits excellent anti-diarrheal properties. Cloves have certain antibacterial effect and immune enhancement, while Ficus hirta Vahl plays a significant role in enhancing physical fitness and immune function. Therefore, the combination of them should indeed be effective in terms of antibacterial activity. At the same time, it can reduce some side effects by regulating the intestinal flora. As a traditional Chinese medicine formula for treating *H. pylori* infection, its antibacterial ability remains the focus of this study, and research on immune enhancement will be published separately.

The purpose of this study was to investigate the anti-*H. pylori* activity of the water extract of HZQYF and explore its potential mechanism of action.

## 2 Materials and methods

### 2.1 Chemicals and reagents

The reagents and materials were obtained as follows.

Sterile-defibrinated sheep blood (Hongquan Biotechnology Co., Ltd., Guangzhou, Guangdong, China). Fetal bovine serum (FBS) (Inner Mongolia Jin Yuan Kang Bioengineering Co., Ltd., Inner Mongolia, China). Brain heart infusion (BHI), MUELLER-HINTON (MH)AGAR and Columbia agar base (Oxiod Ltd. in Basingstoke, Hampshire, UK). Clarithromycin (CLR) and metronidazole (MET) (Macklin Biochemical Co., Ltd., Shanghai, China). Amoxicillin (AMO) (National Institutes for Food and Drug Control, Beijing, China). Levofloxacin (LEF) (Target Molecule Corp., Boston, MA, USA). Phosphate buffered saline (PBS) (Cytiva, Shanghai, China). Purelink RNA kit and BCA protein assay kit (Thermo Fisher Scientific, USA). SYBR Premix Ex Tap^™^ kit and PrimeScript RT reagent kit with gDNA Eraser (Takara Biotechnology Co., Ltd., Minamikusatsu, Japan). Water (Sangon Biotech, Shanghai, China). Urea and acetohydroxamic acid (Shanghai McLean Company, Shanghai, China). N-Phenyl-1-naphthylamine (Aladdin Bio-Chem Technology Co., LTD., Shanghai, China). Tween-20 (Biotopped, Beijing, China). Phenol red (Sigma). Electron microscope fixative (Servicebio, Beijing, China). Chebulic acid, chebulanin, and ellagic acid (Shandong Bokang Jingxi Chemical Co., Ltd., Liaocheng, China). Gallic acid and corilagin (National Institutes for Food and Drug Control, Beijing, China). 4% Paraformaldehyde Fix Solution, DAPI Staining Solution, Antifade Mounting Medium, RIPA reagent, PMSF, 1% protease inhibitor cocktail, SDS-PAGE Gel Preparation Kit, BeyoColor^™^ Prestained Color Protein Marker, BeyoECL Plus Kit, and the second antibodies (1:2500) against mouse (A0216) (Beyotime Institute of Biotechnology, Shanghai, China). FITC (Biotopped Life sciences, Beijing, China). Roswell Park Memorial Institute (RPMI) 1640 (Gibco-Life Technologies LLC., Rockville, USA). Anti-*H. pylori* CagA (sc-28368) (Santa Cruz Biotechnology, Texas, USA).

### 2.2 Plant materials and sample preparation

Chebulae Fructus and Ficus hirta Vahl (Root) were purchased from Guangzhou Zhining Pharmaceutical Co., Ltd. Cloves (flower bud) was purchased from Guangzhou Junyuan Chenxiangshan Traditional Chinese Medicine Herbal Pieces Co., Ltd.

The preparation of HZQYF follows the water extraction method. Firstly, Chebulae Fructus, Ficus hirta Vahl, and Cloves were separately ground and passed through an 80-mesh sieve. 10 g of HZQYF (Ficus hirta Vahl:Chebulae Fructus:Cloves = 6:3:1) were weighed precisely and 10–12 times of water in volume was added. Then the mixture was extracted three times at 90°C for 1 h each time, and the suspension was filtered to obtain the supernatant. The supernatant was subjected to rotary evaporation (N-1300, EYELA, Japan) at 0.08 MPa and 55°C. Afterwards, the concentrate was freeze-dried into a powdered form, and stored at −20°C for future use.

### 2.3 Composition of the herbal extract

Components in the extracts of HZQYF were identified by UHPLC–MS/MS (Thermo scientific). 1 mg/mL of HZQYF solution was prepared in methanol–water (15:85, v/v) and injected into LC–MS system after filtered through a 0.22-μm membrane filter (Sartorius). The LC–MS analysis was performed on an ACQUITY UPLC BEH C18 column (Waters, 2.1*100 mm,1.7 μm) at 30°C. The mobile phase was water containing 0.2% acetic acid (solvent A) and methanol (solvent B) and pumped at a flow rate of 0.2 mL/min. The gradient elution was as follows: 0–5 min, 5–15% mobile phase B; 5–10 min, 15–25% mobile phase B; 10–20 min, 25–60% mobile phase B; 20–35 min, 60–90% mobile phase B; 35–45 min, 90% mobile phase B; 45.1–60 min, 5% mobile phase B. Mass spectrum was performed via an ESI source. Master scan was Full MS monitoring, scan range of mass-to-charge ratio was 100–1,500. The spray voltage was set at 3.5 kV in positive ion mode and 3.0 kV in negative ion mode, ion transfer tube temperature at 325°C and vaporizer temperature at 300°C.

Three batches of the HZQYF were ultrasonically dissolved in ultrapure water to obtain solutions with a concentration of 200 μg/mL individually. A mixed standard containing 0.2 mg/mL of chebulic acid, 0.2 mg/mL of gallic Acid, 0.2 mg/mL of corilagin, 0.08 mg/mL of chebulanin, 0.6 mg/mL of ellagic acid was prepared. The standard and samples were filtered through a 0.22-μm membrane filter before injected into HPLC. The HPLC analysis was performed with a Chromeleon Workstation (UltiMate 3000, USA). Reverse phase chromatography was used under gradient conditions with an Accalim C18 column (Thermo, 4.6 mm × 250 mm, 5-μm) at 25°C. The wavelength of 270 nm was chosen as the detection wavelength according to the UV absorption curve. The mobile phase was water containing 0.1% trifluoroacetic acid (solvent A) and acetonitrile (solvent B). The gradient was composed of 3 to 30% (B) from 0 to 40 min. The flow rate was 1.0 mL/min and the volume injected was 20 μL. The detected compounds were identified by comparing their retention time with those of the standard. The contents of the compounds were expressed as micrograms per gram of extracts by correlating the peak areas with those of the reference substances.

### 2.4 *Helicobacter pylori* strains and growth conditions

The standard strains of *H. pylori,* ATCC 43504, ATCC 700392, and SS1, were obtained from the American Type Culture Collection (ATCC) in Manassas, Virginia, USA. Clinical isolated strains, ICDC111001 and CSO1, were obtained from Guangzhou University of Chinese Medicine and University of Shanghai for Science and Technology, respectively. Clinical isolated strains, QYZ-001, QYZ-003, and QYZ-004, were obtained from Qingyuan Hospital of Traditional Chinese Medicine in Guangzhou.

All clinical strains were maintained at −80°C in a combination of 65% BHI, 25% glycerol and 10% FBS (v/v/v), and confirmed by the providers through morphological inspection, Gram staining, and biochemical reaction. The European Committee on Antimicrobial Susceptibility Testing’s (EUCAST 2019) breakpoint values for antibiotic resistance have been developed. For the current study, all strains were cultured in Columbia agar base supplemented with 5% sterile defibrinated sheep blood at 37°C in a tri-gas incubator (CB170, BINDER, Germany) containing 10% CO_2_, 5% O_2_, and 85% N_2_. As a result of prior whole-genome random sequencing, annotation, and re-annotation of the *H. pylori* ATCC 700392 strain genomes, it was chosen for RT-qPCR detection and transcriptome analysis. Additionally, it is a reference strain that is resistant to none of the tested antibiotics, hence it was also utilized in checkerboard analysis and drug resistance studies.

### 2.5 Minimum inhibition concentration (MIC) and minimum bactericidal concentration (MBC)

The most frequently used method micro-broth dilution and agar dilution method were used in this study to detect the MIC.

Ninety-six-well (96-well) microtiter plates with 50 μL in each well were prepared with twofold serial dilutions of the test substances. In order to achieve a final concentration of 1 × 10^6^ CFU/mL, a 2-day-old *H. pylori* solid culture was obtained in BHI with 20% FBS and inserted into the 96-well microtiter plates with 50 μL in each well. The plates were incubated for 3 days at 37°C in microaerophilic environment with constant 150 rpm shaking. The plates were examined optically after incubation, and the lowest level that produced no turbidity was defined as MIC. Each batch of HZQYF was also tested with clarithromycin as a quality control measure. Additionally, growth controls without any test chemicals and negative controls using HZQYF dissolved in blank medium without microbes were required. The experiments were performed three times. Following the measurement of MIC values using the broth microdilution method, the MBC was established. In short, 100 μL of a solution containing 1, 2, or 4 times the MIC of HZQYF were withdrawn from the 96-well microtiter plate and cultivated for 3 days at 37°C in a microaerophilic environment. A 99.9% decline in viability when compared to the control group was defined as the MBC value. This experiment was conducted three times.

Different *H. pylori* strains were adjusted to 2 McF using DensiCHEK Plus densicheck (BioMerieux, French) and then 100 μL of them were added to MH blood plate that contain different concentrations of HZQYF (80, 160, 320 μg/mL). After 72 h’s cuture, the MICs were defined by the concentration of HZQYF when there was no colony growth on the surface of the blood plate or there was a significant decrease in the number of colonies. And clarithromycin was used as the positive control in each experiment.

### 2.6 Inhibiting kinetics and killing kinetics assays

Assays for killing kinetics and inhibiting kinetics were carried out as follows. *H. pylori* ATCC 43504 and ATCC 700392 were subjected to water (control), CLR (for ATCC 43504 is 0.016 μg/mL and for ATCC 700392 is 0.004 μg/mL) and the HZQYF extracts (40, 80, 160 μg/mL) in BHI broth containing 10% FBS while being shaken at 150 rpm in the tri-gas incubator for the inhibitory kinetics experiments. Then, 100 μL of each sample was pipetted for absorbance measurement at 600 nm using POLARstar Omega (BGM Labtech) at 0, 8, 24, 32, 46, 48, 50, 52, 56, and 72 h. This experiment was repeated three times.

*Helicobacter pylori* ATCC 43504 and ATCC 700392 were inoculated in BHI broth with 10% FBS as part of the killing kinetics experiments. Each sample had 50 μL withdrawn for a series of 10-fold dilutions after being exposed to water (control) or the HZQYF extracts (320, 640, 1,280 μg/mL) for 0, 12, 24, 36, 48, 60, or 72 h. Following that, single colonies were created by plating 100 μL dilutions for 5 days on solid agar. Automatic Colony Counter (count 60, China) was used to count the colonies, and the findings were represented as Log (CFU/mL). This experiment was repeated twice.

### 2.7 Combination with antibiotics

According to a known approach with minor modifications, the effects of HZQYF extracts coupled with antibiotics were assessed. To figure out if HZQYF extracts and antibiotics work together synergistically to treat *H. pylori* infection, six serial, two-fold dilutions of HZQYF extracts were made. The antibiotics included were clarithromycin, metronidazole, amoxicillin, and levofloxacin. A total of 60 μL of *H. pylori* ATCC 700392 bacterium suspension was placed in each well of a 96-well plate, along with 30 μL of HZQYF (0, 160, 320, 640, 1,280, 2,560 μg/mL) extracts and 30 μL of antibiotics to achieve a final bacteria concentration of around 1 × 10^6^ CFU/mL. The concentration ranges of CLR, LEF, MTZ and AMO were from 0 to 0.008 μg/mL, 0 to 0.16 μg/mL, 0 to 12.8 μg/mL, and 0 to 0.4 μg/mL, respectively. The plates were then incubated for 3 days at 37°C, in the same gas atmosphere as described before and shaken at 150 rpm. The fractional inhibitory concentration index (FICI) was created to assess the interactions between HZQYF and antibiotics.

FICI = MIC (A combined)/MIC (A alone) + MIC (B combined)/MIC (B alone).

FICI ≤0.5 was considered synergistic, 0.5 < FICI ≤1 was additive, 1 < FICI ≤2 was indifferent, and FICI >2 was antagonistic. This experiment was repeated three times.

### 2.8 RT-qPCR for detection of virulence gene expression

To collect enough bacteria, *H. pylori* ATCC 700392 was adjusted to 1 McF using DensiCHEK Plus densicheck (BioMerieux, French) and cultured for 2 days. In a second experiment, 1 mL of bacterial solution (OD_600_ = 0.5–0.6) was diluted 50-fold in BHI broth adding 10% FBS with or without the MIC of HZQYF for 24 h. The sample’s total RNA was extracted using Purelink RNA kit. Their concentrations were determined using a NanoDrop 2000 spectrophotometer (Thermo Scientific, USA). On a Thermo Scientific 7,500 Fast Real-Time PCR System (Quantitative Real-Time PCR System), RT-qPCR was carried out using the SYBR Premix Ex Tap^™^ kit (Takara) and following the manufacturer’s instructions. The 2^−ΔΔCt^ method was used to examine the data. Table 1 lists the particular primers used (Shu et al., 2016) to amplify mRNA fragments. The expression regulation of these genes was assessed in comparison to bacteria from the control group following normalization with the reference gene 16S.

**Table 1:** Primers used in RT-qPCR analysis.

Gene	Forward Primer (5′–3′)	Reverse Primer (5′–3′)
*16S*	CCGCCTACGCGCTCTTTAC	CTAACGAATAAGCACCGGCTAAC
*flaA*	ATTGGCGTGTTAGCAGAAGTGA	TGACTGGACCGCCACATC
*flaB*	ACATCATTGTGAGCGGTGTGA	GCCCCTAACCGCTCTCAAAT
*babA*	TGCTCAGGGCAAGGGAATAA	ATCGTGGTGGTTACGCTTTTG
*alpA*	GCACGATCGGTAGCCAGACT	ACACATTCCCCGCATTCAAG
*alpB*	ACGCTAAGAAACAGCCCTCAAC	TCATGCGTAACCCCACATCA
*ureE*	TCTTGGCTTGGATGTGAATG	GGAATGGTTTGAAACGAGGA
*ureF*	GGTCCTGCTGATGGCACTA	GCGTCGTTAGAAGCGTTACG

### 2.9 Scanning electron microscope (SEM)

The impacts of HZQYF on *H. pylori* morphology and ultrastructure were studied using SEM, as previously described (Shen et al., 2021). Initially, in order to grow enough bacteria, *H. pylori* ATCC 700392 was diluted to 1 McF in BHI including 10% FBS and cultured for 24 h under microaerophilic conditions with shaking at 150 rpm. In a second experiment, 1 mL of bacterial solution (OD_600_ = 0.5–0.6) was diluted 50-fold in BHI broth adding 10% FBS with or without the MIC dosage of HZQYF extract for 24 h. After centrifuging the bacteria for 10 min at 6000 rpm, they underwent two PBS washes. They were then left to cure for the night at 4°C in 2.5% glutaraldehyde. Before being lyophilized and fixed, the materials underwent a graded ethanol sequence of dehydration. The specimens were examined using a Sigma500 scanning electron microscope (ZEISS, Germany) after metal spraying.

### 2.10 The impact of cell membrane permeability

The determination of the penetration ability of HZQYF on the outer membrane of *H. pylori* was done by NPN uptake method. *H. pylori* was scraped from blood agar plates and adjusted to an optical density of 1 McF using DensiCHEK Plus densicheck (BioMerieux, French). Different concentrations of HZQYF extracts (80 and 160 μg/mL) were added, and a growth control was set. After incubation in a tri-gas incubator for 24 h, the samples were centrifuged at 6000 rpm for 10 min and washed twice with PBS buffer. Then, they were resuspended in PBS. The bacterial solution and 40 μM NPN were added into a 96-well plate, and incubated at 37°C for 30 min before conducting fluorescence measurements. The fluorescence measurements are performed using a multifunctional microplate reader with an excitation wavelength of 350 nm, an emission wavelength of 420 nm, and an excitation bandwidth of 5 nm. All measurements are repeated three times.

### 2.11 Measurement of urease enzymatic activity

The method of urease determination in this study were based on a previous study with slight modification (Debowski et al., 2017). The strains cultured on blood plates for 48 h were scraped, the turbidity was adjusted to 1 McF using DensiCHEK Plus densicheck (BioMerieux, French), and the same volume of 80 μg/mL and 160 μg/mL aqueous extracts of HZQYF and the bacterial solution were added 1:1 in 6-well plates. A growth control group and a positive control group (40 μg/mL and 80 μg/mL of acetohydroxamic acid) were set up and placed into the triple-air incubator for 24 h at 150 rpm. The collected bacterial solution was centrifuged at 6000 rpm for 10 min, washed twice with PBS, resuspended in 1 mL of PBS after centrifugation, and adjusted to OD_600_ = 0.2. Then, 50 μL of the bacterial suspension was combined with 50 μL of buffer B (pH 6.8, 25 mM phosphate buffer, 0.2% Tween-20). The diluted suspension was placed into a 96-well plate and diluted with 150 μL of buffer C (pH 6.8, 25 mM phosphate buffer, 250 M phenol red), which was then incubated at 37°C for 5 min. A POLARstar Omega (BGM Labtech) plate equipment was used to measure the absorbance at 560 nm every 72 s for 75 cycles after adding 75 μL of a urea solution (0.5 mM) to the wells. The activity was measured as a percentage of the urease activity of the growth control strain and was reported as the rate of change in absorbance over time. The experiment was performed at least three times, and all urease activity measurements were made in duplicate.

### 2.12 Effect on cagA protein expression

In order to detect the expression of the *H. pylori* virulence protein cagA after drug treatment, 1 McF *H. pylori* strains (ATCC 43504 &ATCC 700392) were added into 6-well plates and exposed to water (control) and the HZQYF extracts (80, 160, 320 μg/mL) in BHI broth containing 10% FBS, shaking at 150 rpm in the tri-gas incubator for 24 h. Then, the bacteria of each well were collected after centrifuged at 6000 rpm for 10 min using centrifuging (HERMLE, Germany) and washed twice with PBS. Next, each sample was lysed using 120 μL RIPA reagent containing 1 mM PMSF and 1% protease inhibitor cocktail for bacterial extracts. The protein concentration of the lysate was determined utilizing a BCA protein assay kit. Subsequently, 30 μg of the proteins were loaded onto SDS-PAGE gels for separation and transferred to PVDF membranes. Thereafter, membranes were blocked using 5% skim milk powder for 1 h at room temperature and then blotted with anti-*H. pylori* CagA primary antibodies at 4°C overnight. After that, the membranes were incubated with second antibodies (1:2500) against mouse for 1.5 h at room temperature. Finally, the protein bands were visualized by BeyoECL Plus Kit and exposed with a ChemiScope 6200 (Bio-Rad, Hercules, CA, USA). The IntDen values of the bands were analyzed by ImageJ software.

### 2.13 Effect on adhesion ability

This method refers to Jia’s previous studies (Jia et al., 2022). The GES-1 cells (4 × 10^5^ /well) were seeded into 6-well plates for 24 h and then they were treated with different concentrations of HZQYF (0, 80, 160 and 320 μg/mL). 4 h later, they were infected with ATCC 700392 which was stained with 1% FITC, at a MOI of 100 for 8 h. Next, the mixture was fixed with 4% Paraformaldehyde Fix Solution for 20 min after unadhered *H. pylori* were removed. Subsequently, the 4% paraformaldehyde was discarded. And DAPI Staining Solution and Antifade Mounting Medium were added to stain the cells. Finally, fluorescence signals were analyzed using IM-5FLD Fluorescent inverted microscope (OPTIKA, Italy).

### 2.14 Statistics analysis

Experimental results were analyzed with GraphPad 9.0.0 software and presented as mean ± standard deviation (SD). For the statistical analysis, a one-way ANOVA method based on the Kruskal-Wallis test was applied, and a post-hoc test was then conducted. A statistically significant difference was shown by *p* < 0.05.

## 3 Results

### 3.1 Composition of the herbal extract

**Figure 1:**
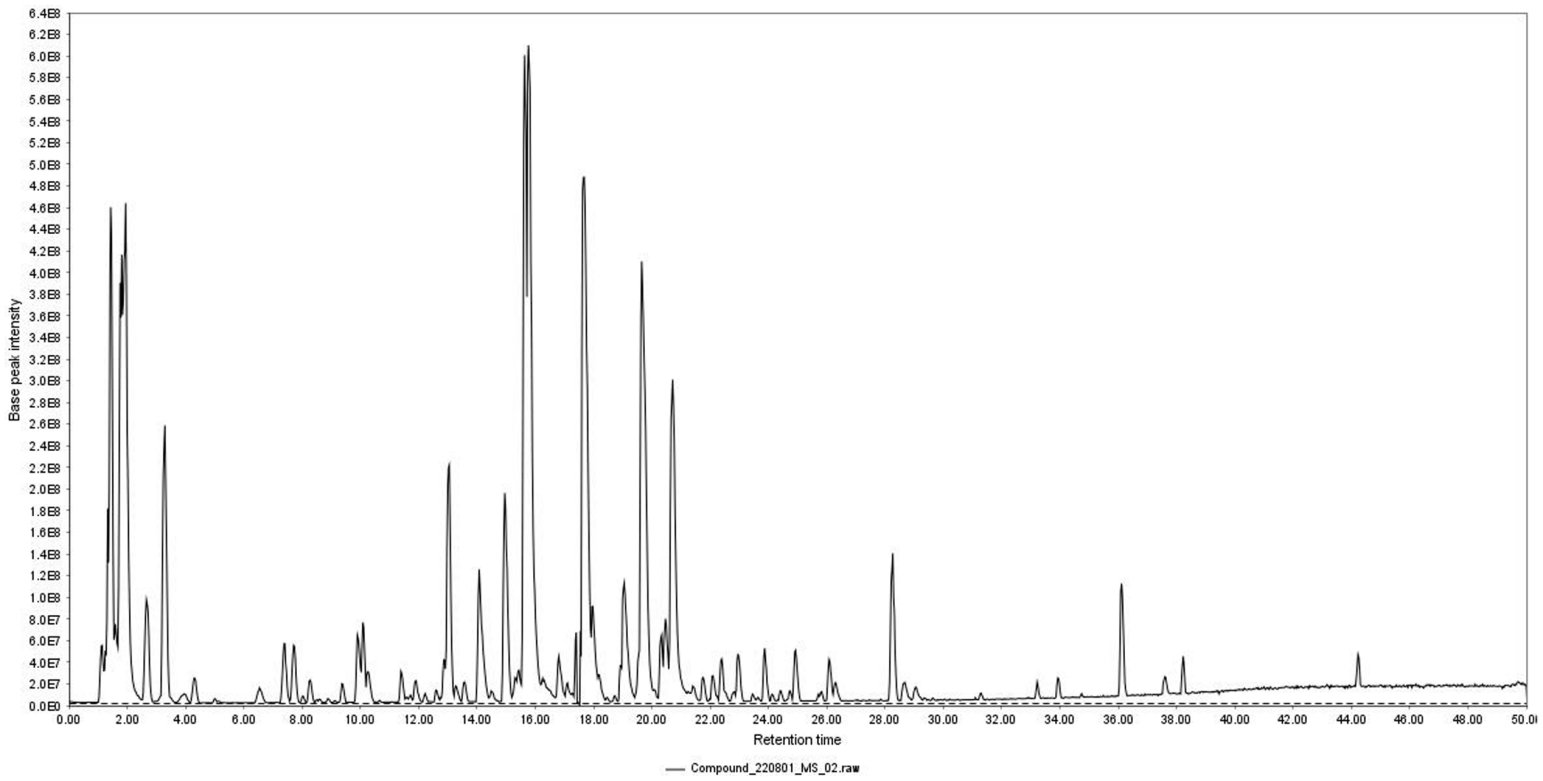
Total ion chromatogram of HZQYF.

Total ion chromatogram of HZQYF was displayed in Figure 1. 31 primary components were identified by UHPLC–MS/MS and listed in Table 2.

**Table 2:** Primary components identified by UHPLC–MS/MS.

No	RT (min)	[M + H]^+^	MS/MS	[M-H]^−^	MS/MS	Molecular	Identification	Errors (ppm)
1	1.60			173.0443	155.0333 [M-H_2_O]^−^	C_7_H_10_O_5_	Shikimic acid	3.96
2	2.19			355.0303	275.0197 [M-2H_2_O-CO_2_]^−^, 293.0288 [M-H_2_O-CO_2_]^−^, 337.0203 [M-H_2_O]^−^	C_14_H_12_O_11_	Chebulic acid	0.44
3	2.35			191.019	111.0073, 87.0073, 85.0280, 129.0180, 191.0549	C_7_H_12_O_6_	Quinic Acid	0.93
4	3.10			781.0533	600.9898 [M-Glu-]^−^, 301.0002	C_34_H_22_O_22_	Punicalin	1.08
5	3.40	171.0287	127.04, 153.02, 135.01	169.0131	97.03, 125.02	C_7_H_6_O_5_	Gallic Acid	3.52
6	4.46			331.0664	125.02, 169.01	C_13_H_16_O_10_	Galloyl-glucose	0.36
7	5.22			343.0667	191.0549, 125.0230	C_14_H_16_O_10_	Galloylquinic acid	0.52
8	9.54			481.0619	300.9953 [M-C_6_H_12_O_6_]^−^	C_20_H_18_O_14_	HHDP-glucose	0.14
9	11.39			483.0773	169.0129, 125.0229, 211.0247, 313.0558	C_20_H_20_O_14_	Digalloyl glucoside	0.37
10	11.45			481.0618	300.9990, 275.0188	C_20_H_18_O_14_	HHDP-hexoside	0.06
11	11.51			481.0619	300.9981, 275.0193, 169.0130, 125.0231	C_20_H_18_O_14_	HHDP-hexoside	0.14
12	11.75			329.08739	167.0336 [M-glucose-H]^−^	C_14_H_18_O_9_	Vanillic acid glucose	0.39
13	12.25			633.0731	300.9983, 275.0192	C_27_H_22_O_18_	Corilagin	0.48
14	14.20	355.1013	193.0489, 259.0597			C_16_H_18_O_9_	Magnolioside	4.57
15	14.46			291.0144	247.0241	C_13_H_8_O_8_	Brevifolin-carboxylic acid	1.06
16	14.98			651.0835	275.0196	C_27_H_24_O_19_	Chebulanin	0.21
17	15.33			635.0888	169.0129, 465.0665, 300.9977, 483.0775	C_27_H_24_O_18_	Trigalloyl-glucoside	0.56
18	15.71			635.08843	465.0684 [M-galloyl-H_2_O]^−^ 313.0557 [M-2galloyl-H_2_O]^−^ 301.0002 331.0669 [M-2galloyl]^−^ 483.0794 [M-galloyl]^−^	C_27_H_24_O_18_	Trigalloyl-glucoside	0.01
19	15.81			633.0728	463.0492, 301.0001	C_27_H_22_O_18_	HHDP-galloyl glucose	0.01
20	17.68			953.0888	300.9984, 463.0477, 633.0623	C_41_H_30_O_27_	Galloyl chebuloyl-HHDP Glucose	0.86
21	18.13			787.0992	635.0907 [M-galloyl]^−^ 483.0786 [M-2galloyl]^−^	C_34_H_28_O_22_	Tetragalloyl glucose	0.25
22	19.66			955.1047	633.0774 [M-Galloyl-gallic]^−^, 275.0199, 337.0196, 319.0079	C_41_H_32_O_27_	Chebulinic acid	0.60
23	20.40	479	303.0491	477.0667	301.0439, 245.0439	C_21_H_18_O_13_	Quercetin-3-o-Glucuronide	0.46
24	20.6	303.013	257.0078, 285.0022, 275.0176, 303.0134			C_14_H_6_O_8_	Ellagic acid	0.89
25	21.75	287.0544	153.0179, 213.0545			C_15_H_10_O_6_	Kaempfero	1.18
26	21.88	463.0562	287.0543	461.0732	285.0399	C_21_H_18_0_12_	kaempferol-3-Oglucuronide	2.59
27	22.0	493.0969	317.0649	491.0828	315.0505	C_22_H_20_O_13_	Quercetin-3-Oglucuronide, 6″-methyl ester	0.46
28	22.60	317.0648		315.0145		C_16_H_12_O_7_	3-Methylquercetin (Isorhamnetin)	4.24
29	22.60	187.03849	115.0543, 143.0491			C_11_H_6_O_3_	Psoralen	1.13
30	23.0	187.0392	186.9813, 186.9968, 187.1096, 187.0951			C_11_H_6_O_3_	Angelicin	1.13
31	24.7	217.0495	174.0612, 202.0256, 203.1275, 217.495, 218.0521			C_12_H_8_O_4_	Bergapte	1.11

**Figure 2:**
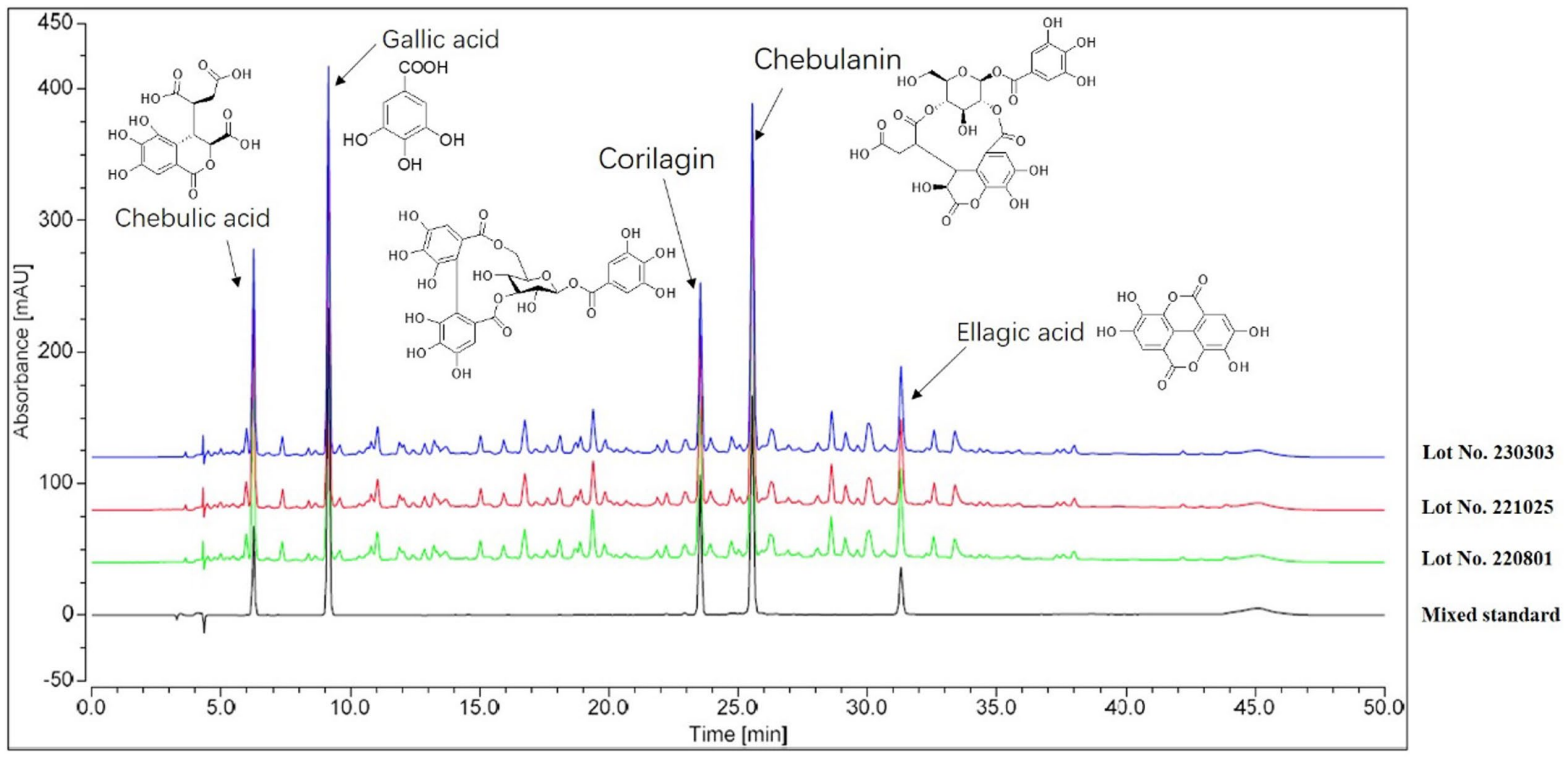
HPLC fingerprint chromatogram of HZQYF.

In order to ensure the consistency of the components in the extracts, three independent batches of HZQYF were separately prepared and then analyzed by HPLC. The HPLC chromatograms of the three separate lots of HZQYF are showed in Figure 2, and the quantitative results are listed in Table 3. The results indicate the composition of the five major components were consistent across various HZQYF batches.

**Table 3:** HPLC quantification of HZQYF.

Compounds	Linearity	*r* ^2^	Concentration range (μg/mL)	Contents in 3 batches (μg/g)		
				220801	221025	230303
Chebulic acid	A = 66.88C + 13.20	1.00	0.9672–19.3440	67.10	66.63	66.31
Gallic Acid	A = 292.91C-23.49	1.00	1.0088–20.1762	30.90	30.65	30.48
Corilagin	A = 161.64C + 4.85	1.00	1.0622–26.5550	27.73	27.48	27.33
Chebulanin	A = 154.37C + 53.75	1.00	1.9784–7.9136	31.77	31.53	31.34
Ellagic Acid	A = 391.04C + 10.10	1.00	0.4245–2.1226	64.06	63.78	63.61

### 3.2 MIC and MBC

The antibacterial properties of HZQYF against multiple strains of *H. pylori* were assessed through the determination of their MIC and MBC. The MIC of HZQYF against three standard *H. pylori* strains and five clinically isolated strains ranged from 80 to 160 μg/mL (Table 4). The MBC of HZQYF against multiple *H. pylori* strains ranged from 160 to 320 μg/mL, indicating that HZQYF exhibited both bacteriostatic and bactericidal effects. In conclusion, these findings demonstrated that HZQYF has the ability to inhibit various strains of *H. pylori*, irrespective of their antibiotic resistance.

**Table 4:** MIC and MBC results of HZQYF on different *H. pylori* strains.

*H. pylori* strains	Drug sensitivity^a^	HZQYF			CLR
		MIC^b^ (μg/mL)	MIC^c^ (μg/mL)	MBC (μg/mL)	MIC (μg/mL)
ATCC 43504	R (MTZ)	160	160	320	0.016
ATCC 700392	S	160	160	320	0.004
SS1	S	160	160	320	0.016
CSO1	R (CLR)	160	160	320	<0.002
QYZ-001	R (MTZ)	160	160	320	< 0.4
QYZ-003	R (CLR, MTZ, LEF)	80	80	160	12.8
QYZ-004	R (CLR, MTZ, LEF, AMO)	80	80	160	1.6
ICDC111001	R (MTZ, LEF)	160	160	320	1.6

^a^ S drug sensitive, R drug resistant. The European Committee on Antimicrobial Susceptibility Testing’s (EUCAST 2019) breakpoint values for antibiotic resistance were established. The drug sensitivity information of these strains has been merged with those stated by Shen et al.

^b^ Using microbroth dilution method.

^c^ Using agar dilution method.

### 3.3 Inhibiting kinetics and killing kinetics assays

**Figure 3:**
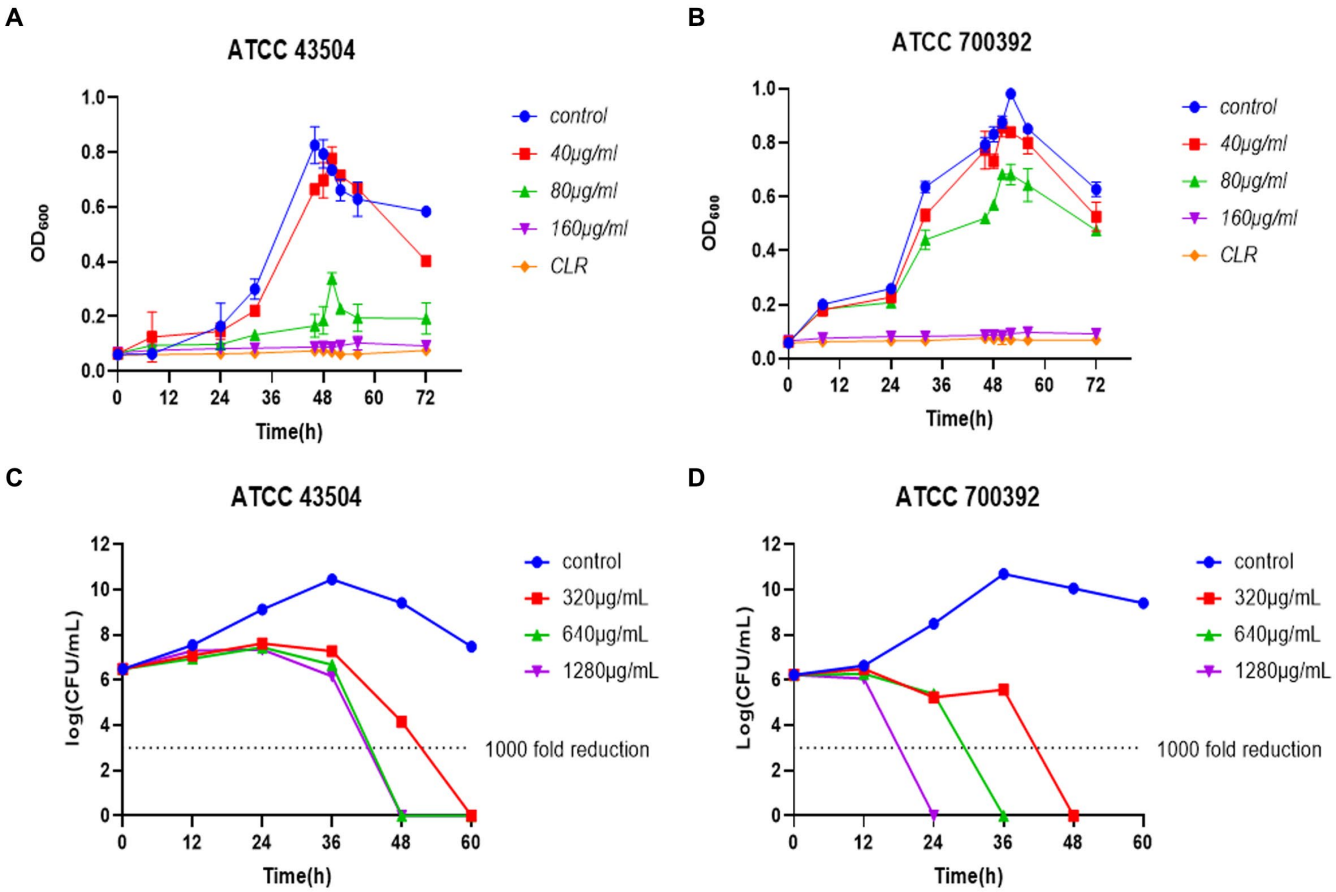
**(A)** Inhibiting kinetics curves of HZQYF on ATCC 43504. Concentration of CLR was 0.016 μg/mL. **(B)** Inhibiting kinetics curves of HZQYF on ATCC 700392. Concentration of CLR was 0.004 μg/mL. **(C)** Killing kinetics curves of HZQYF on ATCC 43504. **(D)** Killing kinetics curves of HZQYF on ATCC 700392.

Based on the findings of the Figures 3A,B, the inhibitory activity of HZQYF on the ATCC 43504 and ATCC 700392 strains exhibited a dose-dependent relationship. In the case of ATCC 43504, a concentration of ½ MIC demonstrated a significant ability to impede bacterial growth, whereas a concentration of ¼ MIC exhibited a mild antibacterial effect on *H. pylori*, primarily characterized by a reduction in the final bacterial count. Regarding ATCC 700392, concentrations of ½ MIC and ¼ MIC displayed slight inhibitory effects on the bacterial strain. However, both MICs can significantly inhibit the growth of the above two bacterial strains, and their inhibitory effects were comparable to clarithromycin.

A time-killing curve was created by counting bacterial colonies at different times. As shown in Figures 3C,D, a concentration of 320 μg/mL (MBC) of HZQYF can completely kill *H. pylori* within 60 h for ATCC 43504, and higher concentrations of 640 μg/mL (2MBC) and 1,280 μg/mL (4MBC) can kill the *H. pylori* within 48 h. HZQYF had a greater bactericidal effect on ATCC 700392 than on ATCC 43504. In the case of ATCC 700392, a concentration of 1,280 μg/mL (4MBC) of HZQYF can eliminate the *H. pylori* within 24 h, 640 μg/mL can eradicate the *H. pylori* within 36 h, and 320 μg/mL (MBC) can eliminate the *H. pylori* within 48 h. The treatments yielded a reduction of over 1,000-fold in the bacterial count at the conclusion of the experiments, in comparison to the initial inoculation.

### 3.4 Effects of HZQYF combined with antibiotics on *Helicobacter pylori*

Using checkerboard determination, the interactions between HZQYF and four antibiotics commonly used in clinical practice for the treatment of *H. pylori* infection were evaluated. As shown in Table 5, the combination of HZQYF with these four antibiotics was not synergistic but also not antagonistic.

**Table 5:** MICs of HZQYF combined with four antibiotics.

*H. pylori*	Combination	MIC (μg/mL)			FICI	Interaction
		A	B	A + B		
ATCC 700392	HZQYF (A) + CLR (B)	160	0.004	160 + 0.001	1.5	Indifferent
	HZQYF (A) + LEF (B)	160	0.4	160 + 0.1	1.25	Indifferent
	HZQYF (A) + MTZ (B)	160	3.2	160 + 3.2	2	Indifferent
	HZQYF (A) + AMO (B)	160	0.1	160 + 0.05	1.5	Indifferent

### 3.5 Effects of HZQYF on the virulence genes expression of *Helicobacter pylori*

**Figure 4:**
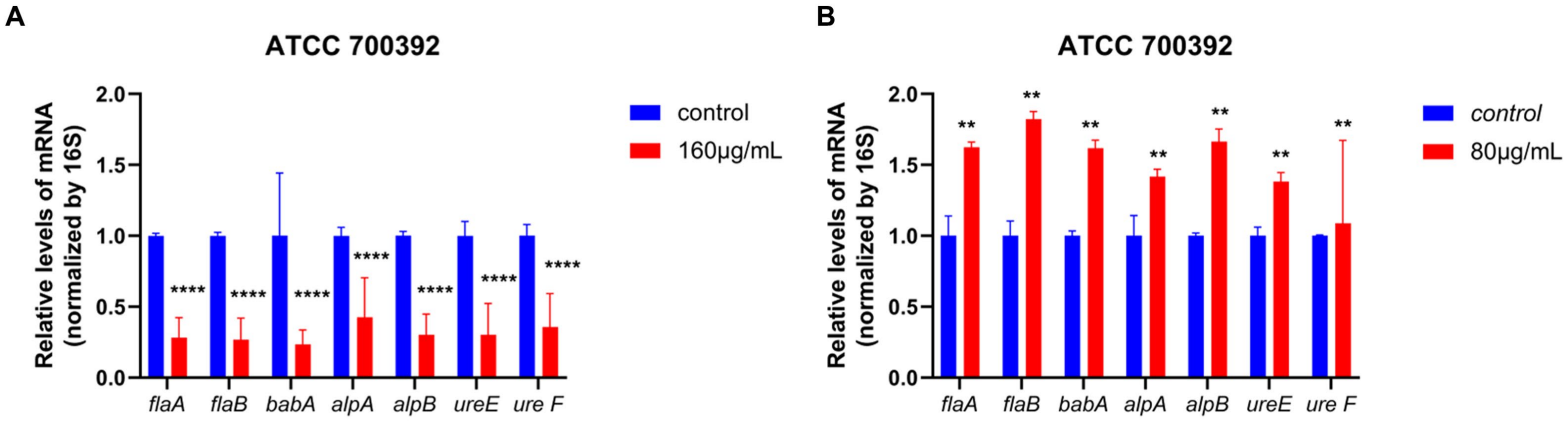
RT-qPCR analysis results showed the effects of HZQYF on the mRNA levels of *H. pylori* virulence genes. The bacteria strain was ATCC 700392, and the concentration of HZQYF were 160 μg/mL **(A)** and 80 μg/mL **(B)**. And the incubation time of HZQYF was all 24 h. *p* < 0.05, vs. control group. **Stands for *p* < 0.01, and ****Stands for *p* < 0.0001, compared with control group.

As demonstrated in Figure 4, for the ATCC 700392 strain, incubation at ½ MIC for 24 h upregulated the expression of flagellar genes (*flaA*, *flaB*), adhesion genes (*alpA*, *alpB*, *babA*), and urease genes (*ureE*, *ureF*). However, at MIC, there was a significant downregulation of flagellar genes (*flaA*, *flaB*), adhesion genes (*alpA*, *alpB*, *babA*), and urease genes (*ureE*, *ureF*) after 24 h of incubation.

### 3.6 Effect of HZQYF on *Helicobacter pylori* morphology

**Figure 5:**
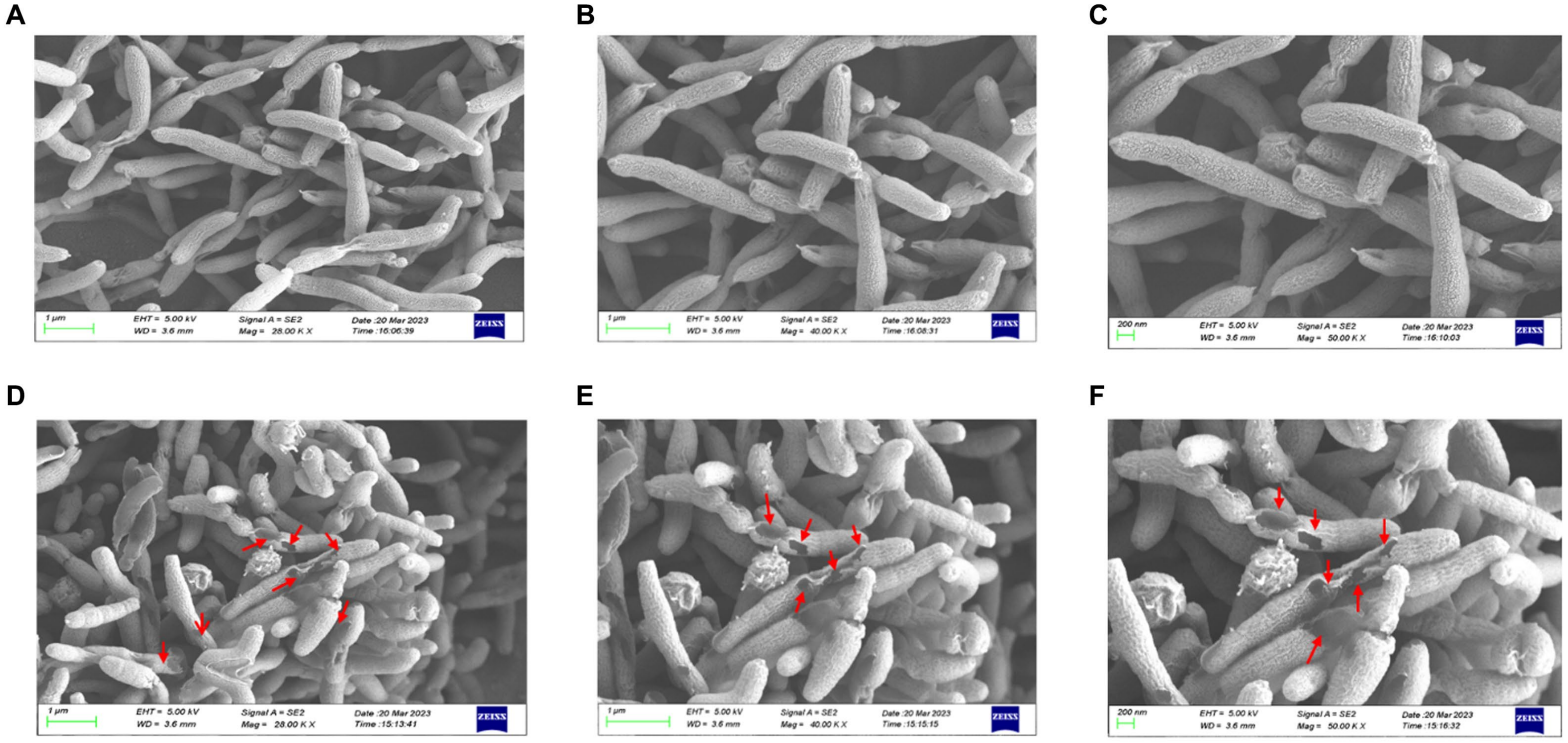
SEM images of ATCC 700392. Morphological images of *H. pylori* cells on SEM (magnification of 20.0, 40.0, and 50.0 kx) of control **(A–C)** and HZQYF treatment **(D–F)** at MIC (160 μg/mL) for 24 h.

Differences in the morphology of *H. pylori* with or without HZQYF under MIC incubation conditions were observed using scanning electron microscopy (SEM). From Figure 5, compared to the control group, ATCC 700392 showed multiple surface damages after 24 h of incubation at MIC. Therefore, HZQYF may disrupt the morphology of bacteria and consequently impact the survival of *H. pylori*.

### 3.7 The impact of cell membrane permeability

**Figure 6:**
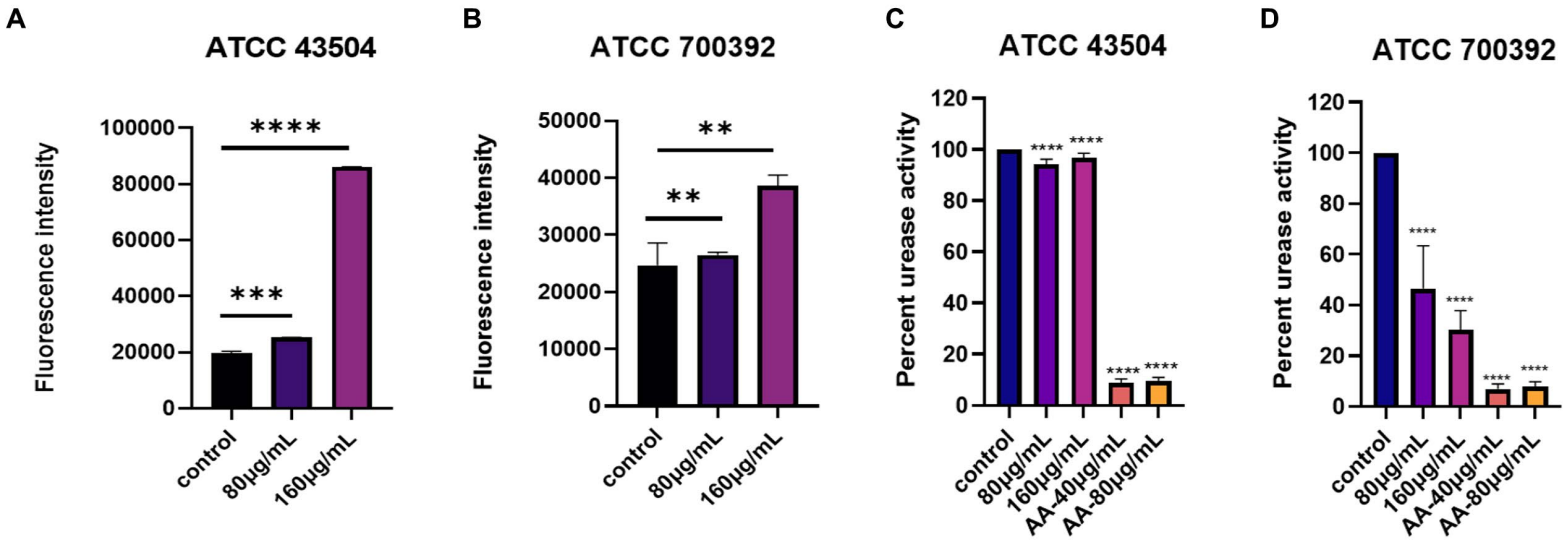
**(A)** The impact of cell membrane permeability of HZQYF on ATCC 43504. **(B)** The impact of cell membrane permeability of HZQYF on ATCC 700392. **(C)** The change in urease activity of ATCC 43504. **(D)** The change in urease activity of ATCC 700392. **Stands for *p* < 0.01, ***Stands for *p* < 0.001, and ****Stands for *p* < 0.0001, compared with control group.

The results of the impact of 1/2 MIC and MIC of HZQYF on the cell membrane permeability of *H. pylori* are shown in Figures 6A,B. Gram-negative bacteria are enveloped by two layers of membranes, the inner membrane and the outer membrane, with the intact outer membrane acting as a permeation barrier. NPN has weak fluorescence in aqueous environments, but strong fluorescence in non-polar or hydrophobic environments. Once the outer membrane structure is damaged, NPN can enter the phospholipid layer, resulting in significant fluorescence. Thus, the ability of *H. pylori* to uptake NPN corresponds to the changes in the structure and permeability of the extracellular membrane. As shown in Figures 6A,B, the fluorescence intensity of intracellular NPN increased proportionally after both ATCC 43504 and ATCC 700392 were treated with HZQYF at ½ MIC and MICs. The fluorescence intensity of the sample-treated group was significantly higher than that of the control group, indicating that the permeability of the outer membrane of *H. pylori* was enhanced after treatment with the HZQYF extracts.

### 3.8 Measurement of urease activity

The change in urease activity of *H. pylori* with different HZQYF concentrations is shown in Figures 6C,D. Urease is one of the key factors for the successful colonization of *H. pylori* in the stomach. Under highly acidic conditions, *H. pylori* can produce a large amount of urease. This urease can hydrolyze urea into ammonia, which can neutralize gastric acid and create an environment suitable for the growth of *H. pylori*. Therefore, inhibiting the activity of urease is an indication of inhibiting the growth of *H. pylori*. As shown in Figure 6, the urease inhibitor acetyl oxime acid (AA, worked as positive control) can significantly inhibit urease activity at 40 μg/mL and 80 μg/mL concentrations for both ATCC 43504 and ATCC 700392 strains. Compared to the control group, HZQYF had an inhibitory effect on urease activity for both ATCC 43504 and ATCC 700392 strains, but the urease activity for ATCC 43504 strain was still 90%, while for ATCC 700392 strain, the urease activity had already decreased to below 50%.

### 3.9 Effect of HZQYF on cagA protein expression using western blot

**Figure 7:**
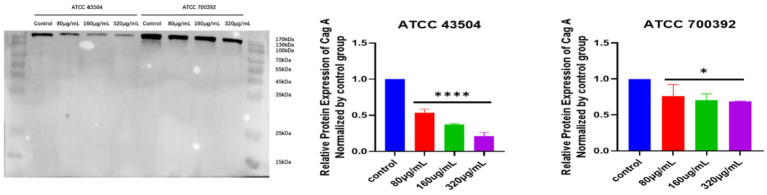
CagA protein expression in *H. pylori* ATCC 43504 and ATCC 700392 in different groups. *Stands for *p* < 0.05, and ****Stands for *p* < 0.0001, compared with control group.

As shown in Figure 7, the expression of CagA proteins of ATCC 43504 and ATCC 700392 were significantly reduced after treatment with HZQYF (80,160, 320 μg/mL) compared with control group.

### 3.10 Effect of HZQYF on adhesion ability

**Figure 8:**
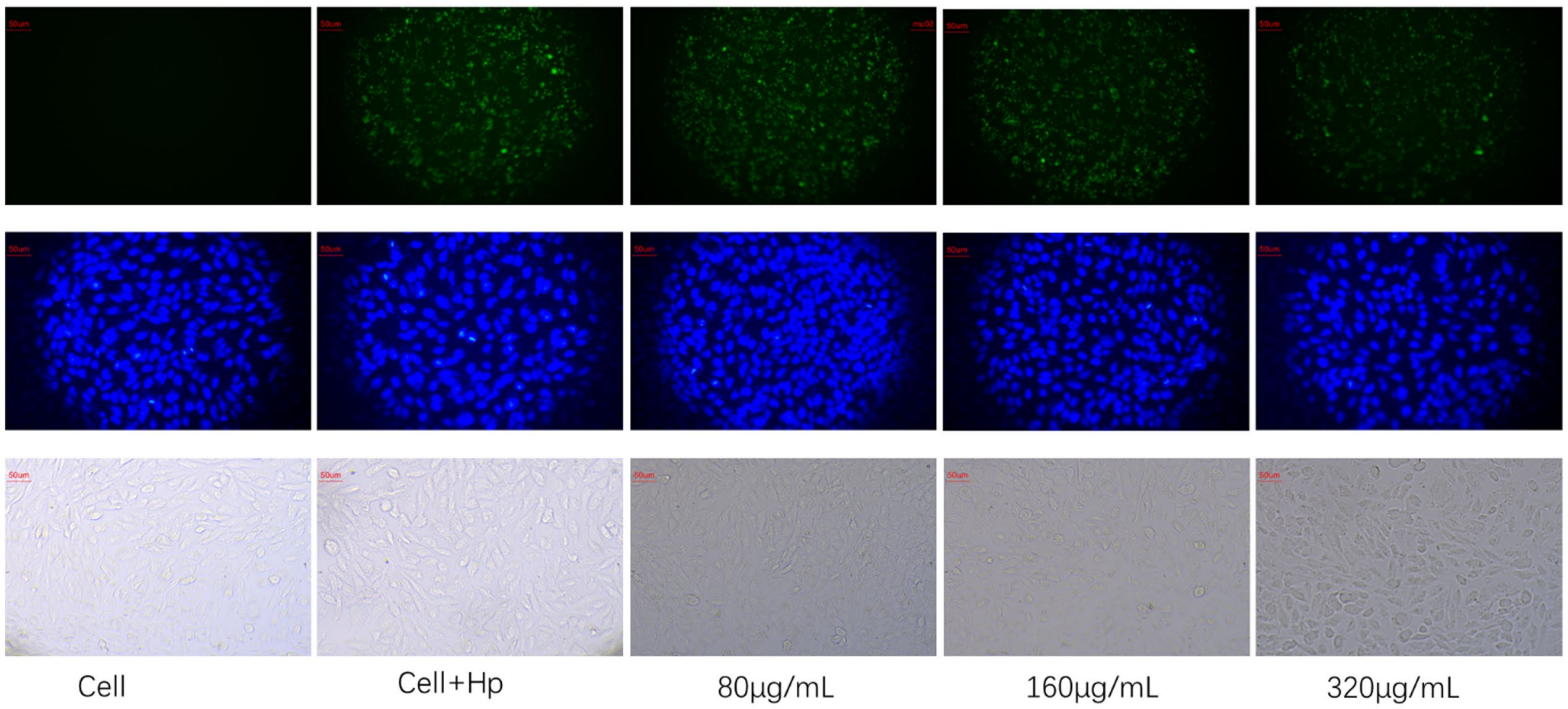
Effect of HZQYF (80,160, 320 μg/mL) on adhesion of ATCC 700392. The first row was marked with 1% FITC for *H. pylori*, and the second row was marked with DAPI, and all groups except cell group were added to *H. pylori* at MOI = 100:1.

As shown in Figure 8, blue fluorescence indicates GES-1 cell nuclei and green fluorescence indicates *H. pylori*. The number of *H. pylori* adhered to GES-1 cells decreased significantly after the administration of HZQYF, and adhesion ability is inhibited gradually with the increase of the dosing concentration.

## 4 Discussion

In this study, a formulation containing Chebulae Fructus, Ficus hirta Vahl, and Cloves was found to have anti-*H. pylori* activity; *in vitro* anti-*H. pylori* activity studies were conducted as well as the preliminary mechanism of action was revealed.

The composition of HZQYF was studied using HPLC. The results showed that the components in three batches of HZQYF were relatively consistent. Among them, the contents of chebulic and ellagic acid were the highest, followed by chebulanin, gallic acid, and corilatin.

The bacteriostatic activity of the HZQYF was confirmed. The MIC and MBC of HZQYF against three standard strains of *H. pylori* and five clinical isolates were studied. The results showed that HZQYF inhibited and killed both standard strains and clinical strains with MICs of 80–160 μg/mL and MBCs of 160–320 μg/mL. The time-inhibition curves showed that HZQYF inhibited *H. pylori* in a dose-dependent manner. The 40 μg/mL-80 μg/mL had a slight inhibitory effect on *H. pylori*, but 160 μg/mL had a strong inhibitory effect on *H. pylori*, which was almost the same as that of the positive control group after clarithromycin treatment. The time-bactericidal curve showed that HZQYF at MIC could control *H. pylori* below 1,000 times of the starting bacterial load at 60 h. If the dose was increased to 640–1,280 μg/mL, *H. pylori* could be controlled below 1,000 times of the starting bacterial load at 48 h.

The effect of HZQYF in combination with antibiotics on *H. pylori* was then investigated. It was found that HZQYF could be combined with clarithromycin, metronidazole, levofloxacin and amoxicillin with neither antagonism nor synergistic.

The mechanism of killing *H. pylori* involved inducing alterations in the visual morphology of the bacterium as well as disrupting the integrity of its cell membrane (Bittencourt et al., 2020). For morphological observations, after 24 h of treatment at the MIC, the SEM results indicated a significant alteration in the morphology of *H. pylori* and interference with the integrity of its cell membrane caused by HZQYF, which might lead to alternations of osmotic pressure and thereby induce bleb formation and cell lysis (Yan et al., 2022).

Previous studies have demonstrated that gastric epithelial adhesion plays a crucial role in enhancing inflammation caused by *H. pylori* (Yonezawa et al., 2016). Specifically, the *alpAB* locus has been identified as encoding an adhesion that is associated with the ability of *H. pylori* to adhere to human gastric tissues (Odenbreit et al., 1999; de Jonge et al., 2004a,b; Lu et al., 2007). In their research, Olga A. Senkovich et al. observed that the deletion of *H. pylori alpA* and *alpB* resulted in a reduction in *H. pylori* binding to laminin. Conversely, the expression of plasmid-borne *alpA* or *alpB* enabled *E. coli* to bind to laminin. Furthermore, *H. pylori* strains lacking *alpB* were also found to lack the ability to bind to laminin. Both *alpA* and *alpB* have been found to play a role in *H. pylori* laminin binding, as demonstrated by the absence of laminin binding in *H. pylori* strains lacking only *alpB*. Furthermore, fucosylated ABO blood group antigens and sialyl-Lewis x/a antigens are believed to function as receptors for *H. pylori* adherence (Boren et al., 1993; Oberhuber et al., 1997). The ABO antigens are specifically recognized by the blood group antigen binding adhesion (*babA*) (Aspholm-Hurtig et al., 2004), while the sialyl-Lewis x/a antigens are recognized by the *H. pylori* sialic acid binding adhesion (Fujimoto et al., 2007).

In our study, we investigated the expression of *alpA*, *alpB*, and *babA* genes using RT-qPCR. The findings revealed a down-regulation in the expression of these genes following the administration of HZQYF. And fluorescent staining was used to detect the effect of *H. pylori* on the adhesion ability on GES-1 cells, and the results showed that HZQYF could inhibit the adhesion ability of *H. pylori*. These may lead to a reduction in long-term infection and the associated inflammatory response.

The inhibition of urease activity is crucial for the eradication of *H. pylori*, as well *H. pylori* as for mitigating excessive oxidative stress and potential cancer development following *H. pylori* infection. Bacterial urease proteins encompass both structural proteins (*ureA*, *ureB*, and *ureC*) and auxiliary proteins (*ureD*, *ureH*, *ureE*, *ureF*, and *ureG*), each playing distinct roles in the activation of urease. The structural proteins form the active center of the urease, while the auxiliary proteins primarily facilitate the delivery of nickel ions (Li et al., 2019). Numerous studies have demonstrated the involvement of urease in the pathogenesis induced by *H. pylori* (Baj et al., 2020), such as overactivation of oxidative stress (Shi et al., 2019), which ultimately induces carcinogenesis (Yang et al., 2021). Therefore, attenuating oxidative stress is particularly important in anti-*H. pylori* therapy. In the current study, the RT-qPCR assay aimed at detecting virulence factors. It was observed that the expression of urease genes *ureE* and *ureF* were down-regulated by HZQYF. These genes primarily contribute to urease activation (Moncrief and Hausinger, 1997; Song et al., 2001; Biagi et al., 2013; Fong et al., 2013; Masetti et al., 2021), leading to the hypothesis that HZQYF might impede the activation process of the urease enzyme, thereby diminishing its activity. In the urease activity assay experiment, it was found that HZQYF could reduce the urease activity of *H. pylori*, but the inhibitory effect on urease varied for different strains of the bacteria. We hypothesized that this might be related to the single nucleotide polymorphisms of different strains of the bacteria. CagA is a Cytotoxin-associated protein which is considered the most important and widely studied virulence factor of *H. pylori*, and associated with gastric mucosal inflammation (Miftahussurur et al., 2020). At present, it has been shown a crucial role in the development of stomach cancer, which may promote the enhancement of drug resistance, proliferation and metastasis, and tumor stemness formation of gastric cancer cells make a difference (Zhang et al., 2012; El-Shouny et al., 2020). It can reduce the expression of cagA protein, which provides a direction for us for further study about effect of HZQYF on related inflammation after *H. pylori* infection.

## 5 Conclusion

In summary, HZQYF has a good bacteriostatic and bactericidal effect on *H. pylori*. There is not antagonistic effect in the use of HZQYF combined with antibiotics. Through scanning electron microscopy, it was observed that after administration, the morphology of *H. pylori* was disrupted, causing damage to its surface. Using the NPN uptake method, it was found that the outer membrane permeability of *H. pylori* was significantly enhanced after administration of the HZQYF. Through RT-qPCR technology, we detected changes in the expression of relevant virulence factor genes. The results showed that after 24 h of cultivation at the MIC, the HZQYF can downregulate the expression of flagellar genes (*flaA, flaB*), urease genes (*ureE, ureF*), and adhesion genes (*alpA, alpB, babA*). It was found through urease activity assays that HZQYF can reduce the urease activity of *H. pylori* and decrease the colonization of *H. pylori*. Based on these results, HZQYF could be an important anti-*H. pylori* agent.

## Data availability statement

The original contributions presented in the study are included in the article/supplementary material, further inquiries can be directed to the corresponding authors.

## Author contributions

ZF: Conceptualization, Data curation, Investigation, Methodology, Project administration, Writing – original draft. HL: Data curation, Formal analysis, Visualization, Writing – original draft. YH: Visualization, Writing – original draft, Writing – review & editing. CP: Data curation, Writing – review & editing. LO: Visualization, Writing – review & editing. JJ: Formal analysis, Writing – review & editing. MX: Data curation, Formal analysis, Writing – review & editing. YZ: Visualization, Writing – review & editing. MC: Visualization, Writing – review & editing. GZ: Conceptualization, Funding acquisition, Writing – review & editing. MY: Conceptualization, Funding acquisition, Investigation, Supervision, Writing – review & editing.

## Glossary

HZQYF: Hezi Qingyou Formula
HPLC: High performance liquid chromatography
RT-qPCR: Reverse Transcription-quantitative Polymerase Chain Reaction
SEM: Scanning electron microscope
NPN: N-phenylnaphthalen-1-amine
MIC: Minimum inhibitory concentration
FBS: Fetal bovine serum
BHI: Brain heart infusion
CLR: Clarithromycin
MET: Metronidazole
AMO: Amoxicillin
LEF: Levofloxacin
PBS: Phosphate Buffered Saline
ATCC: American Type Culture Collection
MBC: Minimum bactericidal concentration
S: Drug sensitive
R: Drug resistant
FICI: Fractional inhibitory concentration index
FITC: Fluorescein Isothiocyanate
DAPI: 2-(4-Amidinophenyl)-6-indolecarbamidine dihydrochloride
